# Identification of co-expression modules and pathways correlated with osteosarcoma and its metastasis

**DOI:** 10.1186/s12957-019-1587-7

**Published:** 2019-03-08

**Authors:** Jian-sheng Wang, Yun-guo Wang, Yong-sheng Zhong, Xue-dong Li, Shi-xin Du, Peng Xie, Gui-zhou Zheng, Jing-ming Han

**Affiliations:** 1 Department of Orthopedics Ward II, Shenzhen Children’s Hospital, Shenzhen, 518000 China; 2 Department of Orthopedics, The Second Hospital of Tianjin Medical University, Tianjin, 300211 China; 3 Department of Neurosurgery, The First Affiliated Hospital of Shantou University Medical College, Shenzhen, 518000 China; 4 Department of Orthopedics, The Third Affiliated Hospital of Shenzhen University Health Science Center, Shenzhen, 518000 China

**Keywords:** Osteosarcoma, Metastasis, Co-expression modules, Pathways

## Abstract

**Background:**

Osteosarcoma is the most common bone tumor that occurs in children.

**Methods:**

To identify co-expression modules and pathways correlated with osteosarcoma and its clinical characteristics, we performed weighted gene co-expression network analysis (WGCNA) on RNA-seq data of osteosarcoma with 52 samples. Then we performed pathway enrichment analysis on genes from significant modules.

**Results:**

A total of 5471 genes were included in WGCNA, and 16 modules were identified. Module-trait analysis identified that a module involved in microtubule bundle formation, drug metabolism-cytochrome P450, and IL-17 signaling pathway was negatively correlated with osteosarcoma and positively correlated with metastasis; a module involved in DNA replication was positively correlated with osteosarcoma; a module involved in cell junction was positively correlated with metastasis; and a module involved in heparin binding negatively correlated with osteosarcoma. Moreover, expression levels in four of the top ten differentially expressed genes were validated in another independent dataset.

**Conclusions:**

Our analysis might provide insight for molecular mechanisms of osteosarcoma.

**Electronic supplementary material:**

The online version of this article (10.1186/s12957-019-1587-7) contains supplementary material, which is available to authorized users.

## Background

Osteosarcoma is an aggressive cancer in the skeletal system that most commonly occurs in children [1]. The proportion of patients who receive a complete clinical response after standard chemotherapy and multimodal treatment of surgery is about 70–75% [2]. For patients diagnosed with metastases, the overall 5-year survival rate was approximately 30%, and for patients with relapsed osteosarcoma, the rate decreased to 15% [3]. Since the development of chemotherapy, survival in localized high-grade osteosarcoma has improved considerably. However, there is still no worldwide consensus on a standard chemotherapy approach [3]. Therefore, exploring the molecular mechanism involved in disease progression and metastasis and its relationship with drug response is critical for identifying effective drugs that overcome drug resistance of tumor.

During these decades, with the dramatic improvement on sequencing technology, the increasing accumulation of sequencing data provides us a data resource to investigate molecular mechanisms of diseases including critical genes, pathways, and networks based on an analysis of omics data. Weighted gene co-expression network analysis (WGCNA) is a method that is widely used in exploration of co-expression modules correlated with traits or phenotypes based on expression data (e.g., expression microarray, transcriptome data) [4]. And the R software package for WGCNA was developed to perform weighted correlation network analysis, including procedures of network construction, module identification, gene selection, calculations of topological properties, data simulation, and visualization [4].

In this study, we performed weighted gene co-expression network analysis based on data from RNA-seq of osteosarcoma to identify critical co-expression modules and pathways correlated with osteosarcoma and its clinical characteristics, which might provide new insights for exploring the underlying molecular mechanisms of osteosarcoma.

## Materials and methods

### Expression profiles of osteosarcoma

Expression profiles of osteosarcoma (GSE87624) were obtained from the Gene Expression Omnibus (GEO) (https://www.ncbi.nlm.nih.gov/geo/). This data included RNA-seq profiles of 52 samples including 44 osteosarcoma tissues, 3 normal bone tissues, 1 osteoblast, and 4 osteosarcoma cell lines based on Illumina HiSeq 2000. After quality control, read mapping, and normalization of read counts of the RNA-seq data, the values of fragments per kilobase million (FPKM) in each gene were calculated.

### Co-expression module analysis of osteosarcoma

Weighted gene co-expression network analysis (WGCNA) was used to investigate co-expression modules related with osteosarcoma and its clinical characteristics. Genes with the top 25% variance of expression values among samples were included in WGCNA. Then sample clustering was performed to detect whether there were outliers in these samples.

After sample clustering, scale independence and mean connectivity analysis of modules with different power values were performed to determine the soft threshold of module analysis. The power value was set from 1 to 20, and then the values of scale independence and mean connectivity were generated according to these power values. The power value was determined when the scale independence value was 0.9. Then co-expression matrix was calculated under the determined power value, with the minimal module size of 30 and the merge cut height of 0.25. A cluster dendrogram among modules and an eigengene adjacency heatmap between modules were generated.

### Module-trait analysis based on clinical characteristics of osteosarcoma

Information on the clinical characteristics of samples with osteosarcoma, including sample type (osteosarcoma/normal), tissue type (osteosarcoma/normal), cell-line type (osteosarcoma/normal), and osteosarcoma type (metastasis/primary/unknown), was collected to identify significant co-expression modules related with the clinical characteristics (as trait). Module-trait relationships were calculated according to the correlation between modules and traits; modules that were significantly correlated with individual traits (*P* value < 0.05, module size < 500) were identified; and genes in significant modules were then exported for further analysis.

### Pathway enrichment analysis of significant co-expression modules

To identify the biological pathways and functions of significant modules, pathway enrichment analysis was conducted by using the R package “clusterProfiler v3.4.4” [5, 6] with genes in significant co-expression modules related with osteosarcoma. Pathways were annotated by information from the Kyoto Encyclopedia of Genes and Genomes (KEGG) database [7] and Gene Ontology (GO) terms [8], and the *P* value was adjusted by the Benjamin-Hochberg methods [9]. Pathways with a *P* value < 0.05 were considered as significant pathways.

### Validation of differentially expressed genes in independent dataset

To validate whether differentially expressed genes identified in GSE87624 were also differentially expressed in other expression datasets, we investigated the expression levels of the top ten differentially expressed genes in another expression dataset (GSE12865), which included 14 samples (12 osteosarcoma tumor samples and 2 normal human osteoblasts as control). Differentially expressed genes of osteosarcoma were analyzed by limma package in R.

## Results

### Expression profiles of osteosarcoma

For GSE87624, FPKM values of 21,884 genes from 52 samples were obtained. Then log2-transformed FPKM values were used for further analysis. Information of clinical characteristics of samples included the sample type (osteosarcoma/normal), tissue type (osteosarcoma/normal), cell-line type (osteosarcoma/normal), and osteosarcoma type (metastasis/primary/unknown). Clinical information for osteosarcoma patients is shown in Table 1.

**Table 1:** Sample information in expression profiling

Type	Number (%)
Osteosarcoma	
Tissues	44 (83.0%)
Cell line	4 (7.5%)
Normal bone	
Tissues	3 (5.7%)
Cell line	1 (1.9%)
Tumor type	
Metastasis	9 (17.0%)
Primary	20 (37.8%)
Unknown	15 (28.3%)

### Co-expression module analysis of osteosarcoma

**Fig. 1:**
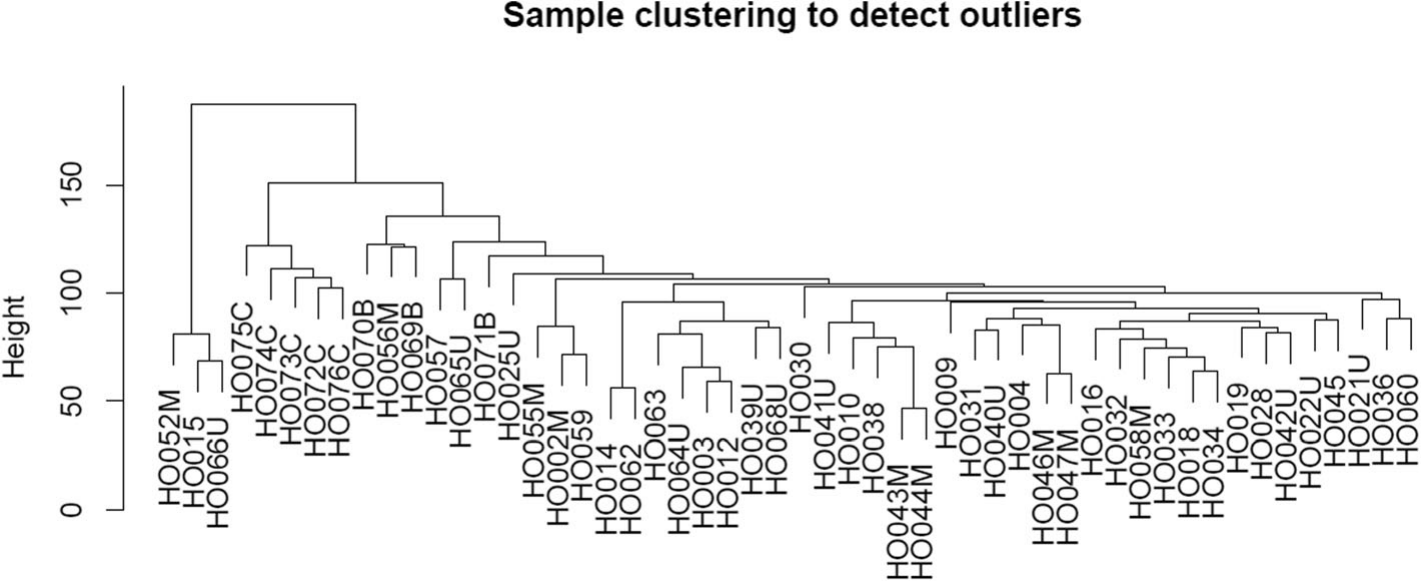
Sample clustering for detecting outliers

By selecting genes with the threshold of the top 25% variance of expression values, a total of 5471 genes were included in WGCNA. As shown in Fig. 1, by sample clustering, no outliers were observed in 52 samples, thus all samples were included in the analysis.

**Fig. 2:**
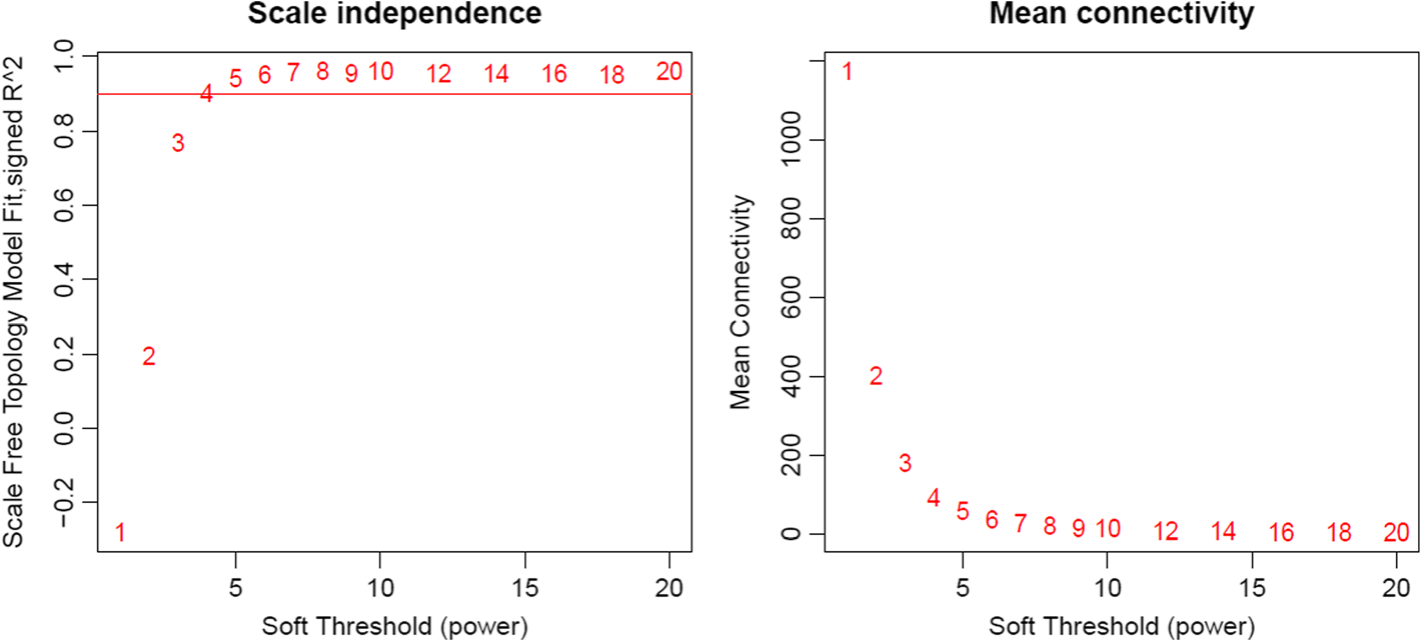
Scale independence and mean connectivity analysis

**Fig. 3:**
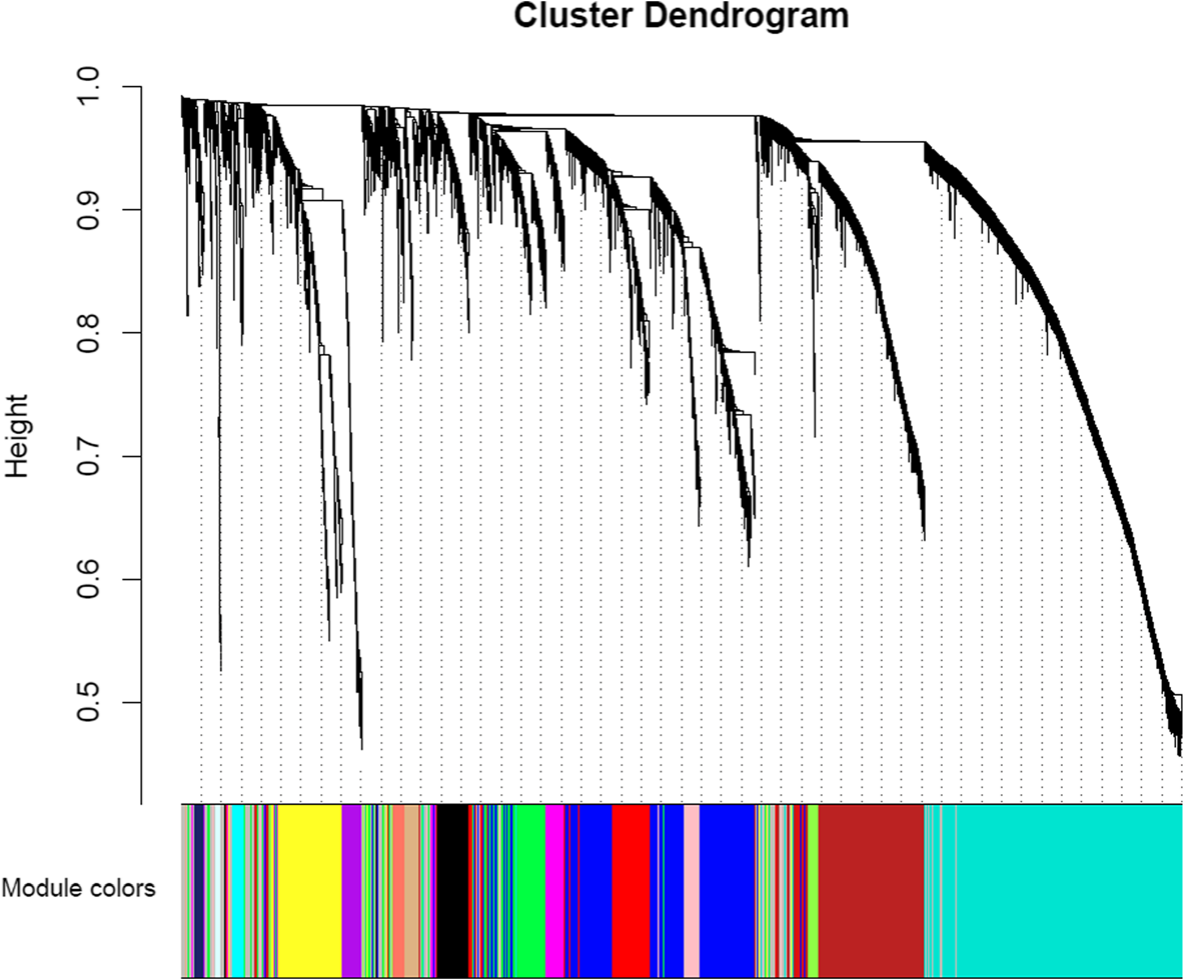
Cluster dendrogram among modules

**Fig. 4:**
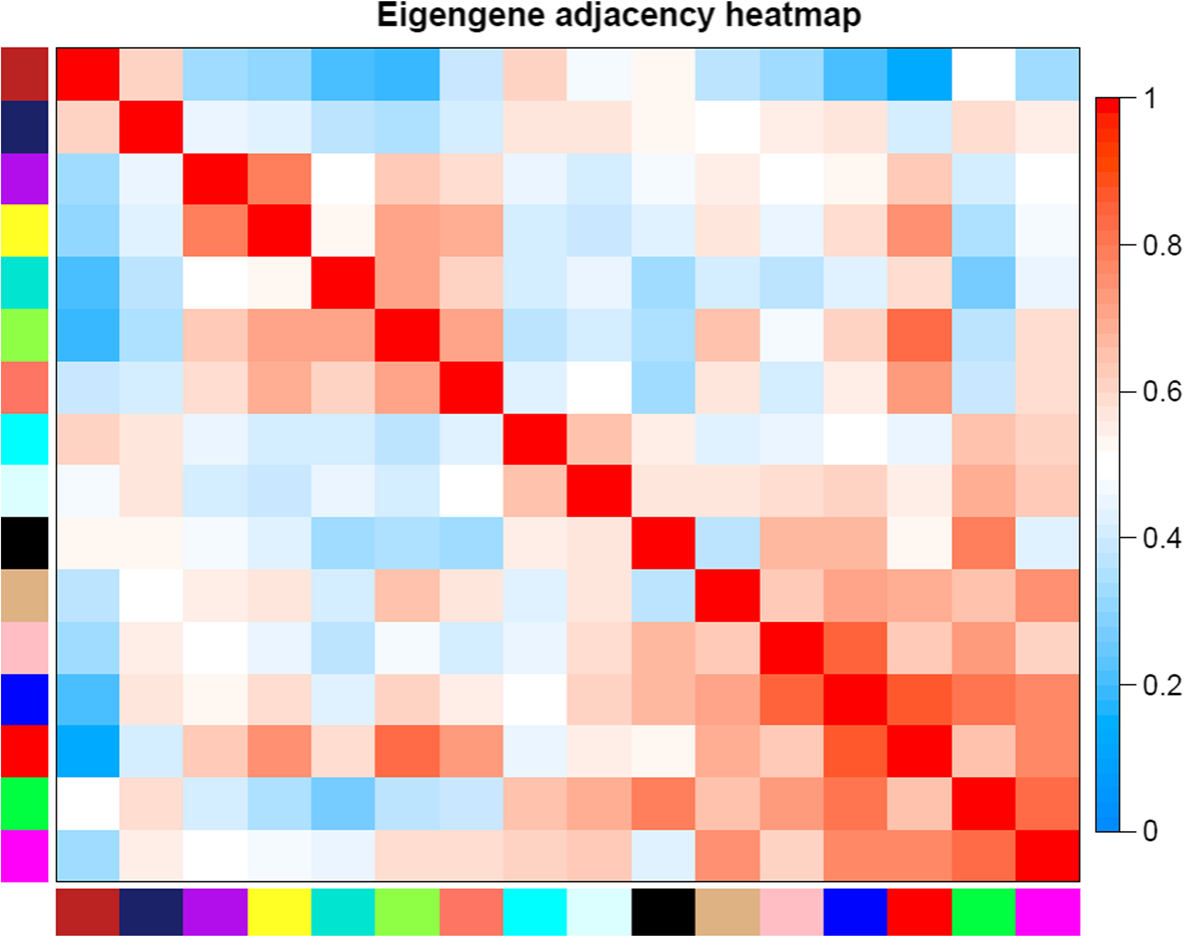
Eigengene adjacency heatmap between modules

Then the soft threshold was determined by scale independence and mean connectivity analysis of modules with different power values ranging from 1 to 20. As shown in Fig. 2, when the power value was set to 4, the scale independence value achieved 0.9 and lower mean connectivity. Therefore, the co-expression matrix was calculated under the determined power value of 4. As shown in Figs. 3 and 4, a total of 16 modules with different genes were generated and displayed with different colors, including 198 genes in a black module, 837 genes in a blue module, 689 genes in a brown module, 60 genes in a cyan module, 408 genes in a green module, 95 genes in a green-yellow module, 31 in a light cyan module, 131 genes in a magenta module, 50 genes in a midnight blue module, 171 genes in a pink module, 127 genes in a purple module, 302 genes in a red module, 63 genes in a salmon module, 88 genes in a tan module, 1413 genes in a turquoise module, and 428 genes in a yellow module.

### Module-trait analysis based on clinical characteristics of osteosarcoma

**Fig. 5:**
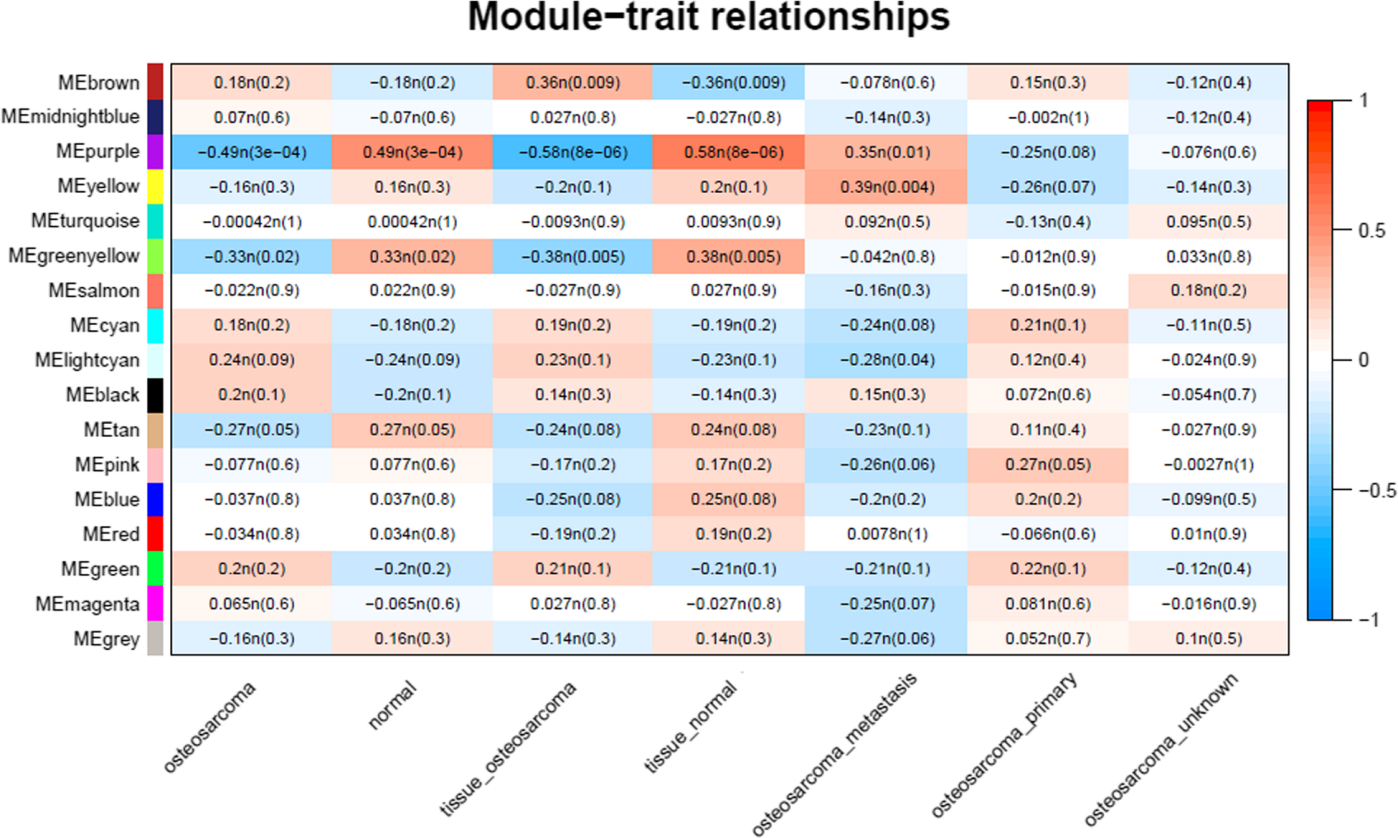
Module-trait relationships

Clinical characteristics of samples with osteosarcoma were collected as a trait, and module-trait relationships were calculated according to the correlation between modules and traits. As shown in Fig. 5, for the sample type of osteosarcoma, the most significant modules with negative correlation were purple and green-yellow; for the tissue type of osteosarcoma, the most significant modules with negative correlation were purple and greenyellow and the positively correlated module was brown; and for the osteosarcoma type of metastasis, the most significant modules with positive correlation were purple and yellow.

### Pathway enrichment analysis

By pathway enrichment analysis, with the threshold of Benjamin-adjusted *P* value < 0.05, we obtained significant GO terms and KEGG pathways enriched in significant modules (see Additional file 1). For the purple module (Additional file 1: Table S1), we identified 22 GO terms and 6 KEGG pathways and microtubule bundle formation, IL-17 signaling pathway, and drug metabolism-cytochrome P450 were identified; for the brown module (Additional file 1: Table S2), we identified 58 GO terms and 3 KEGG pathways, which were mainly related with DNA replication and mitotic nuclear division; for the yellow module (Additional file 1: Table S3), we identified 26 GO terms, which were mainly related with the anchored component of membrane and cell junction; and for the green-yellow module (Additional file 1: Table S4), we identified 3 GO terms and 2 KEGG pathways, which were mainly related with regulation of lipolysis in adipocytes and heparin binding.

### Validation of differentially expressed genes in independent datasets

With the threshold of Benjamin-adjusted *P* value < 0.05 and |log_2_ (fold change)| > 1, we obtained a total of 369 differentially expressed genes of osteosarcoma in GSE87624. The top ten differentially expressed genes were *BPIFA1*, *AGR2*, *MEPE*, *MSMB*, *BPIFB1*, *BGLAP*, *SLPI*, *HBA2*, *LCN2*, and *SERPINB3*. In an independent dataset GSE12865, we observed that all ten genes had the same trend of expression level as that in GSE87624, in which four genes, *MEPE*, *BPIFB1*, *HBA2*, and *SERPINB3*, were also identified to have significantly differential expression levels (shown in Table 2.

**Table 2:** Expression levels of the top ten genes in GSE87624 and GSE12865

Gene	Discovery dataset		Validation dataset	
	Log (fold change)	*P* value	Log (fold change)	*P* value
*BPIFA1*	− 6.93	1.69E−05	− 0.10	5.87E−01
*AGR2*	− 5.77	2.22E−04	− 0.05	8.85E−01
*MEPE*	*− 5.29*	*7.43E*−*03*	*− 3.59*	*8.36E*−*03*
*MSMB*	− 4.70	1.02E−04	− 0.77	6.07E−02
*BPIFB1*	*− 4.64*	*2.61E*−*03*	*− 0.31*	*4.40E*−*02*
*BGLAP*	− 4.59	1.68E−01	− 0.48	3.00E−01
*SLPI*	− 4.24	2.33E−01	− 1.11	1.13E−01
*HBA2*	*− 3.93*	*3.31E*−*02*	*− 1.83*	*7.10E*−*04*
*LCN2*	− 3.82	1.60E−03	− 0.24	3.11E−01
*SERPINB3*	*− 3.76*	*1.17E*−*03*	*− 0.69*	*1.46E*−*02*

## Discussion

In this study, we performed a weighted gene co-expression network analysis (WGCNA) to investigate co-expression modules related with osteosarcoma and its clinical characteristics. Significant modules were identified to be correlated with osteosarcoma. For the purple module, which was mainly related with microtubule bundle formation, drug metabolism-cytochrome P450, and IL-17 signaling pathway and was identified to be negatively correlated with the trait of osteosarcoma, while being positively correlated with the trait of metastasis in osteosarcoma, previous studies have reported that increase of microtubule destabilization was related with G1/G2 phase cell cycle arrest and apoptosis, and microtubule inhibitors could trigger autophagy and cell death in osteosarcoma cell line [10]. Besides, IL-17A/IL-17RA interaction promoted metastasis of osteosarcoma cells [11]. Moreover, the resistance of osteosarcoma to chemotherapy was related to cytochrome P450 3A4 [12]. Our results might provide supporting evidence for these previous findings.

For the brown module, which was mainly involved in DNA replication and mitotic nuclear division and was observed to be positively correlated with the trait of osteosarcoma tissue, previous studies have revealed that genes involved with DNA replication and DNA damage were associated with radiosensitivity of osteosarcoma [13], as well as drug sensitivity [14]. Our results indicated that genes in the brown module might be related with carcinogenesis of osteosarcoma and might provide insights for exploring drug targets for the treatment of osteosarcoma.

For yellow module, which was mainly related with the anchored component of membrane and cell junction and was observed to be positively correlated with the trait of metastasis in osteosarcoma, it has been reported that pathways related with cell junction were involved in metastasis in various tumors, e.g., lung cancer [15], osteosarcoma [16], and pancreatic cancer [17], which might be due to the molecules in cell junction, and the anchored component of membrane felicitated the migration and invasion tumor cells into other tissues and organs [18]. Our results suggest that genes in the anchored component of membrane and cell junction might also play important roles in the metastasis of osteosarcoma and were worthy of further investigation.

For the green-yellow module, which was mainly related with regulation of lipolysis in adipocytes and heparin binding, it was observed to be negatively correlated with the trait of osteosarcoma. In accordance with our results, there were studies showing that heparin could reduce the osteosarcoma proliferation and growth [19, 20], and heparin binding sites might be potential therapeutic targets for osteosarcoma [21].

In the validation section, among the top ten differentially expressed genes in GSE87624, we validated four genes, *MEPE*, *BPIFB1*, *HBA2*, and *SERPINB3*, also had significantly differential expression levels in another expression dataset (GSE12865), demonstrating the results identified from the first dataset can be supported by an independent dataset. For *MEPE*, there were researches revealing its involvement with osteosarcoma [22–24]; for *BPIFB1*, it has been reported that it could inhibit radioresistance in nasopharyngeal carcinoma [25, 26]; for *HBA2*, previous studies have identified different expression levels in the bone marrow of prostate cancer patients [27]; and for *SERPINB3*, it has been identified as mediators of Ras-driven inflammation and oncogenesis [28]. The effect of these genes on osteosarcoma was worthy of further investigation.

## Conclusions

By WGCNA methods on expression data, we identified significant co-expression modules and pathways correlated with osteosarcoma, as well as metastasis of osteosarcoma, and our analysis might provide insights for the molecular mechanisms of osteosarcoma.

## Additional file

Additional file 1: **Table S1.** Significant pathways of the purple module. **Table S2.** Significant pathways of the brown module. **Table S3.** Significant pathways of the yellow module. **Table S4.** Significant pathways of the green-yellow module. (DOCX 27 kb)

## Abbreviations

GEO: Gene Expression Omnibus
GO: Gene Ontology
KEGG: Kyoto Encyclopedia of Genes and Genomes
WGCNA: Weighted gene co-expression network analysis
