# Supplementary material for: Identification of co-expression modules and pathways correlated with osteosarcoma and its metastasis
Source: World J Surg Oncol. 2019 Mar 8;17:46. doi: 10.1186/s12957-019-1587-7 (PMC6408756; doi:10.1186/s12957-019-1587-7)
Supplement: Supplementary file 1 — Table S1. Significant pathways of the purple module. Table S2. Significant pathways of the brown module. Table S3. Significant pathways of the yellow module. Table S4. Significant pathways of the green-yellow module. (DOCX 27 kb) [file 12957_2019_1587_MOESM1_ESM.docx]

Table S1 Significant pathways of purple module

| **ID** | **Gene count** | **P-value** | **FDR** |
| --- | --- | --- | --- |
| GO:0035082~axoneme assembly | 6 | 9.64E-07 | 1.30E-03 |
| GO:0003341~cilium movement | 6 | 1.84E-06 | 1.30E-03 |
| GO:0009913~epidermal cell differentiation | 11 | 3.57E-06 | 1.68E-03 |
| GO:0001578~microtubule bundle formation | 6 | 7.82E-06 | 2.76E-03 |
| GO:0031424~keratinization | 8 | 3.21E-05 | 7.36E-03 |
| GO:0070268~cornification | 6 | 3.49E-05 | 7.36E-03 |
| GO:0030216~keratinocyte differentiation | 9 | 3.93E-05 | 7.36E-03 |
| GO:0008544~epidermis development | 11 | 4.17E-05 | 7.36E-03 |
| GO:0043588~skin development | 10 | 8.28E-05 | 1.30E-02 |
| GO:0003351~epithelial cilium movement | 3 | 1.66E-04 | 2.14E-02 |
| GO:0060271~cilium assembly | 9 | 1.67E-04 | 2.14E-02 |
| GO:0044782~cilium organization | 9 | 2.16E-04 | 2.54E-02 |
| GO:0044458~motile cilium assembly | 3 | 2.55E-04 | 2.77E-02 |
| GO:0031514~motile cilium | 9 | 1.50E-07 | 2.62E-05 |
| GO:0036126~sperm flagellum | 6 | 6.31E-06 | 3.68E-04 |
| GO:0097729~9+2 motile cilium | 6 | 6.31E-06 | 3.68E-04 |
| GO:0097223~sperm part | 8 | 8.56E-06 | 3.74E-04 |
| GO:0031528~microvillus membrane | 3 | 2.36E-04 | 8.26E-03 |
| GO:0044441~ciliary part | 9 | 4.77E-04 | 1.39E-02 |
| GO:0005902~microvillus | 4 | 9.86E-04 | 2.47E-02 |
| GO:0030286~dynein complex | 3 | 2.22E-03 | 4.86E-02 |
| GO:0004745~retinol dehydrogenase activity | 3 | 1.26E-04 | 3.56E-02 |
| hsa00830~Retinol metabolism | 5 | 2.63E-05 | 1.94E-03 |
| hsa00980~Metabolism of xenobiotics by cytochrome P450 | 5 | 4.85E-05 | 1.94E-03 |
| hsa00982~Drug metabolism - cytochrome P450 | 4 | 5.66E-04 | 1.51E-02 |
| hsa05204~Chemical carcinogenesis | 4 | 9.26E-04 | 1.85E-02 |
| hsa04657~IL-17 signaling pathway | 4 | 1.48E-03 | 2.22E-02 |
| hsa05146~Amoebiasis | 4 | 1.67E-03 | 2.22E-02 |

Table S2 Significant pathways of brown module

| **ID** | **Gene count** | **P-value** | **FDR** |
| --- | --- | --- | --- |
| GO:0000070~mitotic sister chromatid segregation | 13 | 1.22E-11 | 2.16E-08 |
| GO:0000079~regulation of cyclin-dependent protein serine/threonine kinase activity | 10 | 6.87E-06 | 1.22E-02 |
| GO:0000082~G1/S transition of mitotic cell cycle | 36 | 1.75E-25 | 3.09E-22 |
| GO:0000083~regulation of transcription involved in G1/S transition of mitotic cell cycle | 10 | 4.41E-08 | 7.81E-05 |
| GO:0000086~G2/M transition of mitotic cell cycle | 18 | 7.23E-06 | 1.28E-02 |
| GO:0000281~mitotic cytokinesis | 11 | 3.20E-08 | 5.68E-05 |
| GO:0000731~DNA synthesis involved in DNA repair | 9 | 2.42E-05 | 4.29E-02 |
| GO:0000732~strand displacement | 8 | 2.50E-05 | 4.43E-02 |
| GO:0000775~chromosome, centromeric region | 20 | 1.89E-14 | 2.65E-11 |
| GO:0000776~kinetochore | 22 | 2.19E-13 | 3.05E-10 |
| GO:0000777~condensed chromosome kinetochore | 25 | 9.23E-16 | 1.24E-12 |
| GO:0000785~chromatin | 17 | 4.78E-08 | 6.67E-05 |
| GO:0000793~condensed chromosome | 8 | 1.45E-05 | 2.02E-02 |
| GO:0000796~condensin complex | 5 | 6.50E-06 | 9.06E-03 |
| GO:0000922~spindle pole | 20 | 4.54E-09 | 6.33E-06 |
| GO:0000940~condensed chromosome outer kinetochore | 6 | 2.33E-06 | 3.24E-03 |
| GO:0003677~DNA binding | 102 | 2.71E-08 | 4.11E-05 |
| GO:0003682~chromatin binding | 45 | 7.50E-12 | 1.14E-08 |
| GO:0003697~single-stranded DNA binding | 15 | 4.05E-06 | 6.15E-03 |
| GO:0003777~microtubule motor activity | 13 | 2.04E-05 | 3.10E-02 |
| GO:0005515~protein binding | 386 | 2.75E-11 | 4.17E-08 |
| GO:0005524~ATP binding | 88 | 1.39E-06 | 2.10E-03 |
| GO:0005634~nucleus | 284 | 8.15E-18 | 1.14E-14 |
| GO:0005654~nucleoplasm | 186 | 4.98E-21 | 6.95E-18 |
| GO:0005657~replication fork | 7 | 1.37E-05 | 1.91E-02 |
| GO:0005737~cytoplasm | 243 | 1.19E-08 | 1.66E-05 |
| GO:0005813~centrosome | 35 | 4.14E-06 | 5.78E-03 |
| GO:0005819~spindle | 22 | 7.60E-10 | 1.06E-06 |
| GO:0005829~cytosol | 186 | 5.58E-13 | 7.78E-10 |
| GO:0005871~kinesin complex | 11 | 1.02E-05 | 1.42E-02 |
| GO:0005874~microtubule | 31 | 3.21E-07 | 4.47E-04 |
| GO:0005876~spindle microtubule | 13 | 1.71E-08 | 2.39E-05 |
| GO:0006260~DNA replication | 42 | 1.10E-24 | 1.95E-21 |
| GO:0006268~DNA unwinding involved in DNA replication | 6 | 1.20E-05 | 2.13E-02 |
| GO:0006270~DNA replication initiation | 18 | 6.02E-17 | 2.00E-13 |
| GO:0006271~DNA strand elongation involved in DNA replication | 8 | 3.44E-07 | 6.09E-04 |
| GO:0006281~DNA repair | 30 | 5.20E-09 | 9.21E-06 |
| GO:0007018~microtubule-based movement | 14 | 5.09E-06 | 9.02E-03 |
| GO:0007051~spindle organization | 9 | 2.41E-08 | 4.26E-05 |
| GO:0007052~mitotic spindle organization | 9 | 7.04E-06 | 1.25E-02 |
| GO:0007059~chromosome segregation | 21 | 1.23E-13 | 2.18E-10 |
| GO:0007062~sister chromatid cohesion | 33 | 6.88E-22 | 1.22E-18 |
| GO:0007067~mitotic nuclear division | 58 | 2.03E-30 | 3.59E-27 |
| GO:0007076~mitotic chromosome condensation | 9 | 1.24E-08 | 2.20E-05 |
| GO:0007080~mitotic metaphase plate congression | 11 | 4.31E-07 | 7.64E-04 |
| GO:0008283~cell proliferation | 35 | 3.22E-07 | 5.70E-04 |
| GO:0010369~chromocenter | 6 | 2.93E-05 | 4.09E-02 |
| GO:0015630~microtubule cytoskeleton | 17 | 1.77E-05 | 2.46E-02 |
| GO:0019901~protein kinase binding | 35 | 4.28E-07 | 6.50E-04 |
| GO:0030496~midbody | 24 | 6.70E-11 | 9.35E-08 |
| GO:0032508~DNA duplex unwinding | 11 | 2.45E-06 | 4.35E-03 |
| GO:0034080~CENP-A containing nucleosome assembly | 12 | 2.10E-07 | 3.72E-04 |
| GO:0034501~protein localization to kinetochore | 6 | 1.20E-05 | 2.13E-02 |
| GO:0042493~response to drug | 34 | 1.19E-08 | 2.10E-05 |
| GO:0042555~MCM complex | 7 | 1.18E-07 | 1.64E-04 |
| GO:0051233~spindle midzone | 9 | 9.42E-08 | 1.31E-04 |
| GO:0051301~cell division | 79 | 1.98E-40 | 3.50E-37 |
| GO:0051726~regulation of cell cycle | 23 | 4.07E-10 | 7.21E-07 |
| hsa03030~DNA replication | 14 | 2.08E-10 | 2.64E-07 |
| hsa04110~Cell cycle | 39 | 2.13E-25 | 2.71E-22 |
| hsa05166~HTLV-I infection | 27 | 2.57E-06 | 3.27E-03 |

Table S3 Significant pathways of yellow module

| **ID** | **Gene count** | **P-value** | **FDR** |
| --- | --- | --- | --- |
| GO:0070062~extracellular exosome | 112 | 1.57E-11 | 4.89E-09 |
| GO:0005886~plasma membrane | 139 | 2.70E-09 | 2.81E-07 |
| GO:0005615~extracellular space | 64 | 2.19E-09 | 3.42E-07 |
| GO:0005887~integral component of plasma membrane | 62 | 8.23E-08 | 5.13E-06 |
| GO:0016324~apical plasma membrane | 24 | 7.11E-08 | 5.55E-06 |
| GO:0042599~lamellar body | 6 | 2.24E-07 | 1.16E-05 |
| GO:0005576~extracellular region | 65 | 6.82E-07 | 3.04E-05 |
| GO:0005923~bicellular tight junction | 13 | 5.09E-06 | 1.99E-04 |
| GO:0045334~clathrin-coated endocytic vesicle | 6 | 1.06E-05 | 3.68E-04 |
| GO:0005578~proteinaceous extracellular matrix | 19 | 1.83E-05 | 5.70E-04 |
| GO:0044267~cellular protein metabolic process | 14 | 1.58E-06 | 3.17E-03 |
| GO:0045121~membrane raft | 15 | 1.36E-04 | 3.27E-03 |
| GO:0016323~basolateral plasma membrane | 14 | 1.28E-04 | 3.33E-03 |
| GO:0016328~lateral plasma membrane | 8 | 1.23E-04 | 3.47E-03 |
| GO:0060441~epithelial tube branching involved in lung morphogenesis | 6 | 1.14E-05 | 7.59E-03 |
| GO:0009952~anterior/posterior pattern specification | 11 | 8.25E-06 | 8.24E-03 |
| GO:0031225~anchored component of membrane | 10 | 6.85E-04 | 1.51E-02 |
| GO:0030054~cell junction | 22 | 8.72E-04 | 1.80E-02 |
| GO:0035115~embryonic forelimb morphogenesis | 7 | 5.43E-05 | 2.69E-02 |
| GO:0031982~vesicle | 10 | 0.001657 | 3.18E-02 |
| GO:0030198~extracellular matrix organization | 15 | 9.31E-05 | 3.67E-02 |
| GO:0009954~proximal/distal pattern formation | 6 | 1.37E-04 | 3.86E-02 |
| GO:0030855~epithelial cell differentiation | 9 | 1.27E-04 | 4.16E-02 |
| GO:0031090~organelle membrane | 8 | 2.51E-03 | 4.51E-02 |
| GO:0016021~integral component of membrane | 136 | 2.67E-03 | 4.52E-02 |
| GO:0009986~cell surface | 23 | 2.96E-03 | 4.75E-02 |

Table S4 Significant pathways of greenyellow module

| **ID** | **Gene count** | **P-value** | **FDR** |
| --- | --- | --- | --- |
| GO:0005811~lipid particle | 7 | 1.60E-07 | 1.39E-05 |
| GO:0008201~heparin binding | 6 | 1.91E-04 | 2.53E-02 |
| GO:0005615~extracellular space | 14 | 8.70E-04 | 3.72E-02 |
